# Pregnancy related hormones increase CYP3A mediated buprenorphine metabolism in human hepatocytes: a comparison to CYP3A substrates nifedipine and midazolam

**DOI:** 10.3389/fphar.2023.1218703

**Published:** 2023-07-05

**Authors:** Muluneh M. Fashe, Taryn A. Miner, John K. Fallon, Amanda P. Schauer, Craig Sykes, Philip C. Smith, Craig R. Lee

**Affiliations:** ^1^ Division of Pharmacotherapy and Experimental Therapeutics, UNC Eshelman School of Pharmacy, University of North Carolina at Chapel Hill, Chapel Hill, NC, United States; ^2^ Division of Pharmacoengineering and Molecular Pharmaceutics, UNC Eshelman School of Pharmacy, University of North Carolina at Chapel Hill, Chapel Hill, NC, United States

**Keywords:** pregnancy, targeted proteomics, CYP3A4, CYP3A5, hepatic metabolism, pregnancy related hormones, buprenorphine, nifedipine

## Abstract

**Introduction:** Pregnancy increases the clearance of CYP3A4 substrate drugs and pregnancy-related hormones (PRHs) induce hepatic CYP3A4 expression and metabolism. However, it remains unclear to what extent the magnitude of PRH-evoked changes in hepatic CYP3A metabolism varies across multiple substrates. This study quantified the impact of PRHs on CYP3A protein concentrations and buprenorphine metabolism in human hepatocytes, and compared the magnitude of these effects to nifedipine and midazolam metabolism.

**Methods:** Sandwich-cultured human hepatocytes (SCHH) from female donors were exposed to PRHs, administered in combination across a range of physiologically relevant concentrations, for 72 h. Absolute protein concentrations of CYP3A4, CYP3A5, and CYP3A7 in SCHH membrane fractions were quantified by nanoLC-MS/MS, and norbuprenorphine (nor-BUP), dehydro-nifedipine (dehydro-NIF), and 1-hydroxy-midazolam (1-OH-MDZ) formation was evaluated.

**Results:** Compared to control, PRH exposure increased CYP3A4, CYP3A7, and total CYP3A protein concentrations, but not CYP3A5 concentrations, and increased nor-BUP, dehydro-NIF, and 1-OH-MDZ formation in a concentration-dependent manner. The formation of nor-BUP, dehydro-NIF, and 1-OH-MDZ each positively correlated with PRH-mediated changes in total CYP3A protein concentrations. The PRH-evoked increase in nor-BUP formation was evident in all donors; however, the PRH induction of dehydro-NIF and 1-OH-MDZ formation was diminished in a hepatocyte donor with high basal CYP3A5 expression.

**Discussion:** These findings demonstrate that PRHs increase buprenorphine, nifedipine, and midazolam metabolism in SCHH via induction of CYP3A4 and total CYP3A protein concentrations, and the magnitude of these effects vary across hepatocyte donors in a substrate-specific manner. These data provide insight into the contribution of PRH induction of CYP3A4 metabolism to increased buprenorphine clearance during pregnancy.

## Introduction

Approximately 80% of pregnant individuals use at least one medication to treat a chronic or acute condition, and about 30% are prescribed multiple medications (Haas et al., 2018). Although the pharmacokinetics and effects of many drugs are altered during pregnancy, most drugs lack pregnancy-specific efficacy, safety, and pharmacokinetic data (Gonzalez et al., 2015; Pariente et al., 2016; Tasnif et al., 2016). As a result, medication selection and dosing in pregnant patients is often based on trial-and-error and provider experience (Couzin-Frankel, 2022). Physiologically based pharmacokinetic (PBPK) modeling studies suggest that pregnancy-mediated changes in hepatic clearance are a key driver of altered systemic exposure for certain medications (Dallmann et al., 2018a; Dallmann et al., 2018b). Similarly, experimental studies in human primary hepatocytes have demonstrated that pregnancy-related hormones (PRHs) such as estradiol (E2), progesterone (P4), and cortisol (CRT) alter the hepatic expression and function of certain drug metabolizing enzymes (DMEs) (Choi et al., 2013; Zhang et al., 2015; Khatri et al., 2021b; Fashe et al., 2022).

Cytochrome P450 3A4 (CYP3A4) is responsible for the clearance of over 50% of drugs that undergo hepatic metabolism, including various commonly prescribed medications during pregnancy such as the opioid buprenorphine (BUP) and antihypertensive nifedipine (NIF) (Rendic and Guengerich, 2015; Tasnif et al., 2016). Opioid use disorder (OUD) and hypertensive disorders of pregnancy (HDP) are common pregnancy-related complications that affect approximately 7% (Hirai et al., 2021) and 13% (Ford et al., 2022) of pregnant individuals, respectively, and the CYP3A substrates BUP and NIF are considered first-line treatments for these conditions (ACOG, 2017; ACOG, 2019). In humans, pregnancy is associated with increased oral clearance and reduced systemic exposure of CYP3A substrates, including the prototypical probe substrate midazolam (MDZ) (Pariente et al., 2016; Tasnif et al., 2016). Probe substrate studies have shown that CYP3A metabolic activity, as measured by CYP3A-mediated metabolite formation and excretion, is significantly increased during pregnancy compared to postpartum (Tracy et al., 2005; Hebert et al., 2008). Similar to MDZ, human pharmacokinetic studies have also reported that BUP and NIF oral clearance significantly increases during pregnancy, which results in decreased drug exposure, frequent treatment failures, and a need for higher doses or more frequent dosing to control opioid withdrawal symptoms and blood pressure, respectively (Prevost et al., 1992; Clark et al., 2015; Bastian et al., 2017; Caritis et al., 2017; Mulrenin et al., 2021). PBPK modeling studies suggest that an increase in hepatic CYP3A4-mediated intrinsic clearance is a key mechanism that facilitates these increases in oral clearance (Ke et al., 2012; Quinney et al., 2012; Zhang et al., 2018). However, it remains unclear to what extent the increases in BUP oral clearance observed during pregnancy in humans are mediated by induction of hepatic CYP3A4 expression and increased norbuprenorphine (nor-BUP) formation.

Consistent with the hypothesis that increased secretion of PRHs drives altered hepatic drug metabolism during pregnancy through transcriptional regulation of DME expression (Jeong, 2010; Isoherranen and Thummel, 2013), experimental studies in human primary hepatocytes have established that PRHs increase hepatic *CYP3A4* mRNA levels, CYP3A4 protein expression, and *in vitro* metabolism of select CYP3A4 substrates including MDZ and NIF (Choi et al., 2013; Zhang et al., 2015; Khatri et al., 2021b). Although similar effects would be expected with the CYP3A substrate BUP, experimental studies directly investigating the impact of PRHs on CYP3A4-mediated metabolism of BUP in human hepatocytes have not been completed to date. In addition, prior studies have not quantified the impact of PRHs on absolute CYP3A4, CYP3A5, and total CYP3A protein concentrations within and across multiple human hepatocyte donors, and directly quantified and compared the impact of PRHs on CYP3A-mediated metabolism across multiple clinically relevant substrates within the same experimental system. Therefore, it remains unclear to what degree the magnitude of hepatic CYP3A-mediated metabolism changes during pregnancy vary from substrate to substrate, and are influenced by factors such as basal CYP3A4 and CYP3A5 expression, isoform-specific induction of CYP3A4 relative to CYP3A5, and the relative contribution of CYP3A4 and CYP3A5 isoforms to the metabolic clearance of BUP, NIF, and MDZ.

We hypothesize that PRHs increase the hepatic metabolism of BUP, NIF, and MDZ via induction of hepatic CYP3A4 and total CYP3A protein expression, and the magnitude of these effects will vary by substrate and individual hepatocytes donors. The primary objectives of this study were to 1) quantify the concentration-dependent impact of PRHs on absolute CYP3A4, CYP3A5, CYP3A7, and total CYP3A protein concentrations and the CYP3A-mediated metabolism of BUP in Sandwich-cultured human hepatocytes (SCHH); 2) compare the magnitude of PRH effects on the CYP3A-mediated metabolism of BUP, NIF, and MDZ across multiple hepatocyte donors; and 3) evaluate the relationship between PRH-mediated induction of CYP3A4 and total CYP3A absolute protein concentrations and metabolite formation for each substrate. A secondary objective was to initially investigate the contribution of inter-hepatocyte donor differences in basal CYP3A5 expression to PRH-evoked changes in BUP, NIF, and MDZ metabolism.

## Materials and methods

### Reagents and Chemicals

Reagents were obtained from Life Technologies Corporation (Carlsbad, CA) unless otherwise indicated. Dimethyl sulfoxide (DMSO), rifampin (RIF), MDZ, 1-hydroxy-midazolam (1-OH-MDZ), α-Hydroxymidazolam-d4, NIF, dehydro-NIF, BUP, nor-BUP, norbuprenorphine-d3, estrone (E1), E2, estriol (E3), P4, and CRT were purchased from Sigma-Aldrich (St. Louis, MO). E1, E2, E3, P4, CRT, and RIF stock solutions were prepared in DMSO. Placental growth hormone (pGH) was obtained from R&D Systems (Minneapolis, MN), and stock solution was prepared in pure water. Corning Biocoat™ collagen I coated plates and Matrigel^®^ Matrix were purchased from Corning (Corning, NY). Dehydronifedipine-d6 was obtained from Toronto Research Chemicals (Toronto, ON, Canada). QualGro™ Seeding, QualGro™ Culture, and QualGro™ Induction media were purchased from BioIVT (Durham, NC).

### Sandwich-Cultured Human Hepatocytes

Cryopreserved human primary hepatocytes derived from adult female donors of reproductive age (18–49 years, as defined by the World Health Organization) were purchased from Life Technologies Corporation (Carlsbad, CA) (Hu8339, Hu8373, Hu8375, Hu1970) or BioIVT (YNM). Hepatocyte donor characteristics are summarized in Supplementary Table S1. The hepatocytes were cultured as SCHH as previously discussed (Swift et al., 2010; Fashe et al., 2022). Briefly, hepatocytes were thawed at 37°C, centrifuged (100 x g for 10 min), and resuspended in QualGro™ Seeding medium. The cells were seeded at a density of 250,000 cells/well for proteomics or 150,000 cells/well for metabolism in 24-well and 48-well Corning Biocoat™ collagen I coated plates, respectively. The cells were maintained in 5% CO_2_ at 37°C overnight, and the medium was replaced with QualGro™ Culture media supplemented with 0.25 mg/mL Corning Matrigel^®^ Matrix for 24 h. On days 3–5, the SCHH were treated with vehicle control (0.1% DMSO), RIF (10 µM), or PRH cocktails in QualGro™ Induction medium, as previously described (Fashe et al., 2022).

In each experiment, E1, E2, E3, P4, CRT, and pGH were administered to SCHH as a PRH cocktail in QualGro™ Induction medium during the 72 h induction period. The treatment concentrations and rationale of treatment concentration selection in our experimental model has been presented in detail previously (Fashe et al., 2022), and is summarized in Supplementary Table S2. Briefly, three PRH cocktails, T2, T3, and T3-90th%, were composed of E1, E2, E3, P4, CRT, and pGH at varying concentrations to achieve the concentration of each hormone in culture medium that targeted the average maternal plasma concentration during trimester 2 (T2) and trimester 3 (T3), and the upper range (90th percentile) of T3 plasma concentrations, respectively. The culture medium was replenished twice daily (at 9 a.m. and 5 p.m.) with fresh QualGro™ Induction medium supplemented with the vehicle control or the T2, T3, or T3-90th% PRH cocktails throughout the 72 h exposure period. On day 6, the cells were harvested for membrane protein isolation or exposed to drug substrates for metabolism experiments.

### Membrane protein isolation and quantitative targeted absolute proteomics

The absolute protein concentration of CYP3A4, CYP3A5, and CYP3A7 was determined in the membrane fraction of SCHH exposed to vehicle control, PRH cocktails (T2, T3, and T3-90th%), or RIF. Experiments within each of the five hepatocyte donors were conducted independently and included biological replicates of 3–4 per experimental group. SCHH soluble (cytosol) and insoluble (membrane) fractions were isolated using detergent differential fractionation, as previously described (Qasem et al., 2021; Fashe et al., 2022). Briefly, the cytosolic fraction was removed from the cells using a buffer composed of 0.015% digitonin, 10 mM PIPES, 300 nM sucrose, 100 mM NaCl, 3 mM MgCl_2_, 5 mM EDTA, and 1 M PMSF. After centrifugation at 16,000 × *g* at 4°C for 15 min, the pellet was resuspended in a buffer composed of 0.5% Triton X-100, 10 mM PIPES, 300 mM sucrose, 100 mM NaCl, 3 mM MgCl_2_, 5 mM EDTA, and 1M PMSF, incubated for 30 min in cold-room, and centrifuged at 16,000 × *g* at 4°C for 15 min. The supernatant was collected as the membrane fraction, and protein concentration was determined using a Bio-Rad Protein Assay Kit II (Hercules, CA).

Absolute protein concentrations of CYP3A4, CYP3A5, CYP3A7, and CYP2C8 were quantified in SCHH membrane fractions using a previously described quantitative targeted absolute proteomics (QTAP) isotope dilution nanoLC-MS/MS method (Fallon et al., 2013; Khatri et al., 2019; Fashe et al., 2022). Briefly, the SCHH membrane fraction (20 µg) in 0.5% Triton X-100 was evaporated, resuspended in sodium deoxycholate (1%), and mixed with 0.5 or 1 pmol stable isotope labeled (SIL) peptides (CYP3A4: LSLGGLLQPEKPVVLK; CYP3A5: DTINFLSK; CYP3A7: EIDTVLPNK; CYP2C8: NLNTTAVTK) (purchased from JPT, Berlin, Germany). This was followed by digestion with trypsin, stopping the reaction with low pH, and centrifugation at 13,000 × *g* for 5 min to remove precipitated deoxycholate. Phase extraction of the peptides in the supernatant was performed using Phenomenex Strata X 33u Polymeric Reversed Phase Cartridges and eluted with acetonitrile/0.1% formic acid (ACN/FA 3:2). The samples were dried by evaporation and resuspended in ACN/0.1%FA (1:49), and transferred to LC inserts after brief centrifugation at 13,000 x g for 5 min. The peptides were separated in a Waters nanoAcquity (Waters, Milford, MA) BEH130 C18 column (150 μm × 100 mm, 1.7 μm particle size) and analyzed in a SCIEX QTRAP 5500 hybrid mass spectrometer (Framingham, MA). The LC solvents were A) 0.1% FA/ACN (99:1) and B) ACN, and the elution was achieved as previously described (Khatri et al., 2019). Chromatogram visualization and analysis were performed using Skyline 21.1 software (Pino et al., 2020). The absolute protein concentrations of CYP3A4, CYP3A5, and CYP3A7 in each sample were determined from the peak area ratio of analyte to SIL, normalized to total membrane protein content, and presented as pmol/mg as previously described (Khatri et al., 2021b; Fashe et al., 2022). Since total CYP3A activity is considered as the sum activity of the CYP3A isoforms (Kuehl et al., 2001), the absolute concentration of total CYP3A protein in each sample was quantified by calculating the sum total of absolute CYP3A4, CYP3A5, and CYP3A7 concentrations.

### Buprenorphine, nifedipine, and midazolam metabolism and LC-MS/MS analysis

The metabolism of BUP, NIF, and MDZ was studied in SCHH from four of the five hepatocyte donors (Hu8339, Hu8373, Hu8375, and Hu1970) following exposure to vehicle control or PRH cocktails (T2, T3, and T3-90th%). A sufficient number of cells from donor YNM were not available to conduct metabolism experiments for each substrate. Experiments within each hepatocyte donor were conducted independently with biological replicates of 3–4 per experimental group. At the end of the 72 h induction period, the culture medium was carefully removed and SCHH were washed with William’s E Medium. SCHH were then incubated with William’s E Medium containing either BUP (15 µM for 1.5 h), NIF (25 µM for 1 h), or MDZ (2.5 µM for 1 h), at 37°C. The substrate concentrations approximated previously reported *Km* values in human liver microsomes (Patki et al., 2003; Picard et al., 2005), and substrate incubation duration was selected based on preliminary experiments. At the end of the incubation period, the medium (250 µL) and cell lysates (in 250 µL 70% ACN) were collected and stored at −80°C prior to metabolite quantification by LC-MS/MS. In pilot experiments, the mean concentration of nor-BUP in SCHH cell lysate constituted over 20% of total metabolite formed. Thus, nor-BUP concentration was quantified in both cell lysate and culture medium in each experiment, and the total (cell + media) concentration in each well was calculated and used for data analysis. In contrast, we previously reported that dehydro-NIF concentrations in SCHH lysate constituted a minor fraction (<5%) of total metabolite formed (Khatri et al., 2021b). In pilot experiments, the mean concentration of 1-OH-MDZ in SCHH cell lysate also constituted a minor fraction (<10%) of total metabolite formed. Thus, dehydro-NIF and 1-OH-MDZ metabolite concentrations were quantified solely in SCHH media in each experiment.

#### Nor-BUP quantification

Quantification of nor-BUP in SCHH culture medium and cell lysate was completed by modifying a previously published assay (Marin and McMillin, 2016). Briefly, 100 µL SCHH media or cell lysate (in 70% ACN) was added to 200 µL ACN (0.1% FA) supplemented with 1 μg/mL nor-BUP-d3, centrifuged at 14,000 rpm for 20 min, and then 150 µL was transferred to LC-MS inserts. A 10 µL sample was injected to a Phenomenex Kinetex^®^ 2.6 µm EVO C18 100 Å, LC Column 50 × 2.1 mm (Torrance, CA) coupled to a Thermo TSQ Quantum Ultra triple quadrupole mass spectrometer for separation and subsequent detection. The flow rate was 0.7 mL/min and gradient elution was achieved in LC-MS solvents: A) 0.1% FA in water, and B) ACN (0.1% FA). The nor-BUP lower limit of quantification (LLOQ) was 7.8 ng/mL. The calibration standard and quality control samples precision and accuracy were within 20%.

#### Dehydro-NIF quantification

Dehydro-NIF was quantified in SCHH culture medium as previously described (Khatri et al., 2021b). Briefly, 25 µL media was added to 150 µL methanol supplemented with dehydro-NIF-d6 (internal standard), mixed, and centrifuged. Then, 25 µL supernatant was further diluted with 150 µL water prior to injection into a Waters Atlantis T3 (50 mm × 2.1 mm, 3 μm particle size) analytical column coupled to an AB SCIEX 5000 triple quadruple mass spectrometer for chromatographic separation and subsequent detection. Chromatographic separations were achieved under gradient elusion using 0.1% FA in water and 0.1% FA in ACN. Dehydro-NIF LLOQ was 40.0 ng/mL, and calibration standard and quality control samples precision and accuracy were within 20%.

#### 1-OH-MDZ quantification

Quantification of 1-OH-MDZ in SCHH medium was completed as previously described (Lee et al., 2022). Briefly, media samples (225 µL) were added to an equal volume of ACN supplemented with the SIL internal standard α-OH-MDZ-d4 (0.1 µM), vortexed to mix, and centrifuged at 14,000 rpm for 20 min. The supernatant (400 µL) was collected in a fresh tube, evaporated in vacuum to dryness, and reconstituted in 200 µL 0.1% FA and ACN/methanol at a 95:5 ratio prior to quantification of 1-OH-MDZ by LC-MS/MS. A 10 µL sample was injected into a Phenomenex Kinetex^®^ 2.6 µm EVO C18 100 Å (50 × 2.1 mm) analytical column (Torrance, CA) coupled to a Thermo TSQ Quantum Ultra triple quadruple mass spectrometer (Thermofisher, Waltham, MA) for chromatographic separation and subsequent detection, respectively. The flow rate was 0.5 mL/min, and gradient elution was achieved using LC-MS solvents: A) 0.1% FA in water and B) 0.1% ACN/Methanol (95:5). The LLOQ for 1-OH-MDZ was 0.34 ng/mL. Precision and accuracy of the calibration standards and quality control samples were within 20%.

### Data analysis

Data are presented as mean ± standard deviation (SD) absolute protein concentration, metabolite concentration, or fold-change relative to vehicle control, unless otherwise indicated. Since experiments within each hepatocyte donor were carried out independently, the absolute protein (CYP3A4, CYP3A5, CYP3A7, total CYP3A) and metabolite (nor-BUP, dehydro-NIF, 1-OH-MDZ) concentration data were first analyzed across biological replicates within each donor. Within each hepatocyte donor, the PRH cocktail induced fold-difference in absolute protein concentration or metabolite formation in each sample was calculated by dividing the quantified concentration of each sample by the mean concentration of the vehicle control group (*n* = 3–4 replicates). Then, the mean fold-difference in protein concentration or metabolite formation was calculated for each experimental group (T2, T3, T3-90th%) within each hepatocyte donor. Finally, the mean fold-difference data within each hepatocyte donor in each experimental group was carried forward as a single data point for the analysis of the net concentration-dependent impact of PRHs across hepatocyte donors. All non-normal data were log-transformed prior to statistical analysis. Comparisons across PRH cocktail experimental groups and vehicle control were carried out using a one-way analysis of variance (ANOVA). If the ANOVA *p*-value was <0.05, differences across each experimental group were assessed with a *post hoc* Fisher’s LSD test. A Pearson correlation analysis was conducted to evaluate the relationship between CYP3A4 or total CYP3A protein concentrations and metabolite formation across hepatocyte donors. Statistical significance for all analyses was defined as *p* < 0.05. Data analysis was completed using GraphPad Prism 9.2 (GraphPad Software, La Jolla, CA) and Microsoft Excel (Microsoft, WA).

## Results

### Basal expression of CYP3A isoforms in SCHH vary across donors

**FIGURE 1:**
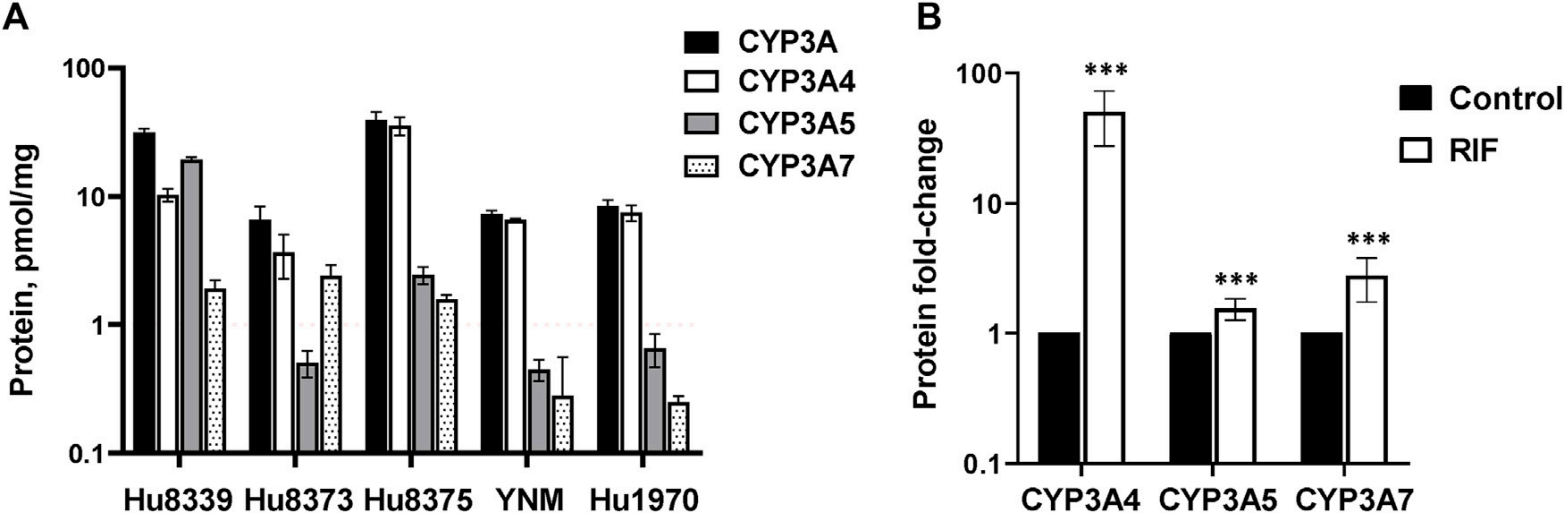
Protein expression of CYP3A isoforms in sandwich-cultured human hepatocytes (SCHH). SCHH (*n* = 5 donors) were exposed to vehicle control or rifampin (RIF) for 72 h (*n* = 3–4 replicates per group). **(A)** Basal absolute protein concentrations of total CYP3A, CYP3A4, CYP3A5, and CYP3A7 in SCHH exposed to vehicle control (0.1% DMSO) by hepatocyte donor. **(B)** Fold-induction of CYP3A isoforms in SCHH exposed to 10 µM RIF relative to vehicle control across all donors (*n* = 5 per group). ***p < 0.001 *versus* vehicle control.

Hepatocyte donor characteristics are summarized in Supplementary Table S1. Quantification of CYP3A4, CYP3A5, and CYP3A7 absolute protein concentrations revealed basal variation in a protein and donor-dependent manner (Figure 1A). Basal concentrations of CYP3A5 (19.2 ± 0.82 pmol/mg) were approximately 2-fold higher than CYP3A4 in donor Hu8339, a CYP3A5 expresser that did not carry the nonfunctional CYP3A5*3 allele. CYP3A4 exhibited the highest protein concentration in all donors that were considered CYP3A5 non-expressers. CYP3A5 concentrations were below 1 pmol/mg in donors Hu8373, YNM, and Hu1970, and was 2.44 ± 0.38 pmol/mg in donor Hu8375. Basal CYP3A4/5 concentrations in our experiments were comparable to previously reported values (Khatri et al., 2019). CYP3A7 concentrations were <2 pmol/mg protein in all donors except Hu8373 (2.4 ± 0.49 pmol/mg). To examine sensitivity of the SCHH model for CYP3A protein induction, CYP3A isoform protein concentrations were quantified following exposure to 10 µM RIF. RIF strongly induced CYP3A4 protein concentrations (50.0 ± 22.6-fold) and mildly induced CYP3A5 (1.55 ± 0.29-fold) and CYP3A7 (2.71 ± 1.06- fold) concentrations (Figure 1B).

### PRHs increased CYP3A absolute protein concentrations in SCHH

**FIGURE 2:**
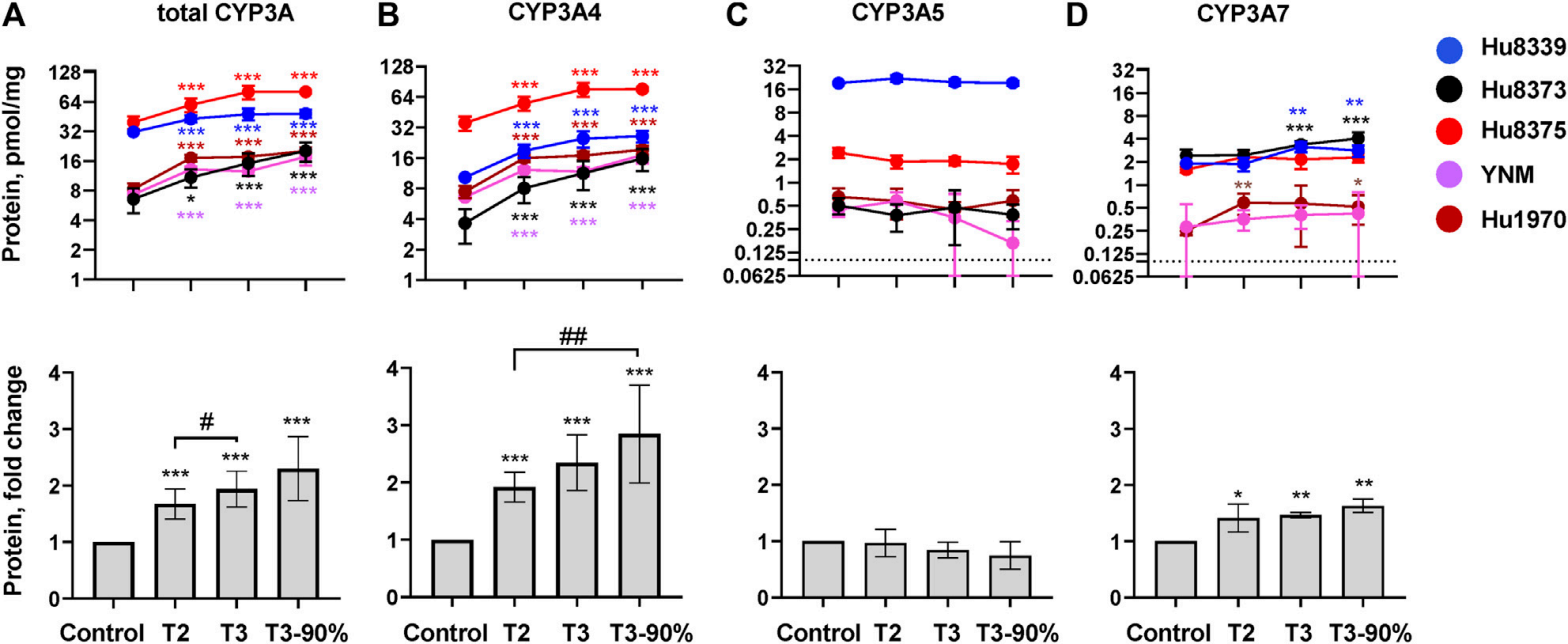
PRHs increased CYP3A4 and total CYP3A protein concentration in sandwich-cultured human hepatocytes (SCHH). SCHH (*n* = 5 donors) were exposed to vehicle control or PRH cocktails targeting average trimester 2 (T2), average trimester 3 (T3), or upper range of T3 (T3-90%) for 72 h. The line graphs depict mean ± SD absolute protein concentration of **(A)** total CYP3A, **(B)** CYP3A4, **(C)** CYP3A5, and **(D)** CYP3A7 within each hepatocyte donor (*n* = 3–4 replicates per group). The dotted line at *y* = 0.1 pmol/mg represents lower limits of quantitation. The corresponding bar graphs exhibit the net mean ± SD fold-difference for each protein relative to the vehicle control across all donors (*n* = 5 per group). *p < 0.05, **p < 0.01, ***p < 0.001 *versus* vehicle control. #Represents the concentration-dependent effect across PRH groups (#< 0.05, ##*p* < 0.01).

The impact of PRHs on the absolute protein concentration of CYP3A4, CYP3A5, CYP3A7, and total CYP3A within each hepatocyte donor, and the net effect across hepatocyte donors, was quantified and compared (Figure 2; Supplementary Table S3). PRHs significantly increased both total CYP3A (Figure 2A) and CYP3A4 (Figure 2B) protein concentrations in SCHH. The PRH-mediated induction of total CYP3A and CYP3A4 protein progressively increased with increasing PRH concentrations and was observed within each of the five hepatocyte donors (ANOVA *p* < 0.001). Relative to the vehicle control, the average net increase in total CYP3A and CYP3A4 protein concentrations across hepatocyte donors was 1.94 ± 0.32-fold and 2.34 ± 0.48-fold, respectively, in the T3 PRH group. The magnitude of these effects within each donor ranged from 1.52 ± 0.21-fold (Hu8339) to 2.31 ± 0.60-fold (Hu8373) for total CYP3A and from 1.79 ± 0.18-fold (YNM) to 3.11 ± 0.98-fold (Hu8373) for CYP3A4 (Supplementary Table S3). In contrast, PRHs did not significantly alter absolute CYP3A5 protein concentration within any individual donor or across all donors (Figure 2C). The PRHs induced a modest net increase in CYP3A7 protein concentrations across donors (ANOVA *p* = 0.008), with a 1.63 ± 0.26-fold increase relative to vehicle control in the T3 group. A PRH-mediated increase in CYP3A7 expression was observed in donors Hu8339, Hu8373, and Hu1970, but no significant impact was observed in donors Hu8375 and YNM (Figure 2D).

The impact of PRH exposure on total CYP3A protein concentrations appeared more pronounced in hepatocyte donors Hu8373, Hu8375, YNM, and Hu1970 (CYP3A5 non-expressers) compared to the CYP3A5 expresser donor Hu8339 (Supplementary Table S3). The PRH-induced changes in total CYP3A and CYP3A4 protein concentrations were compared across CYP3A5 expresser and non-expresser donors (Supplementary Figure S1). Relative to control, the PRH increase in total CYP3A concentrations ranged from 1.76 ± 0.24-fold (T2) to 2.50 ± 0.43-fold (T3-90%) in CYP3A5 non-expressers and from 1.37-fold (T2) to 1.54-fold (T3-90%) in the CYP3A5 expresser donor. In contrast, the PRH induction of CYP3A4 protein concentrations was comparable in CYP3A5 non-expressers and the CYP3A5 expresser.

In addition to CYP3A4, CYP2C8 is a minor contributor to nor-BUP formation *in vitro* (Picard et al., 2005). Hence, we also quantified CYP2C8 absolute protein concentrations (Supplementary Table S4). Although PRHs induced a modest net increase in CYP2C8 protein concentrations across donors (ANOVA *p* = 0.014), a significant PRH-mediated increase in CYP2C8 was only observed in donor Hu8373.

### PRHs increased buprenorphine, nifedipine, and midazolam metabolism in SCHH

**FIGURE 3:**
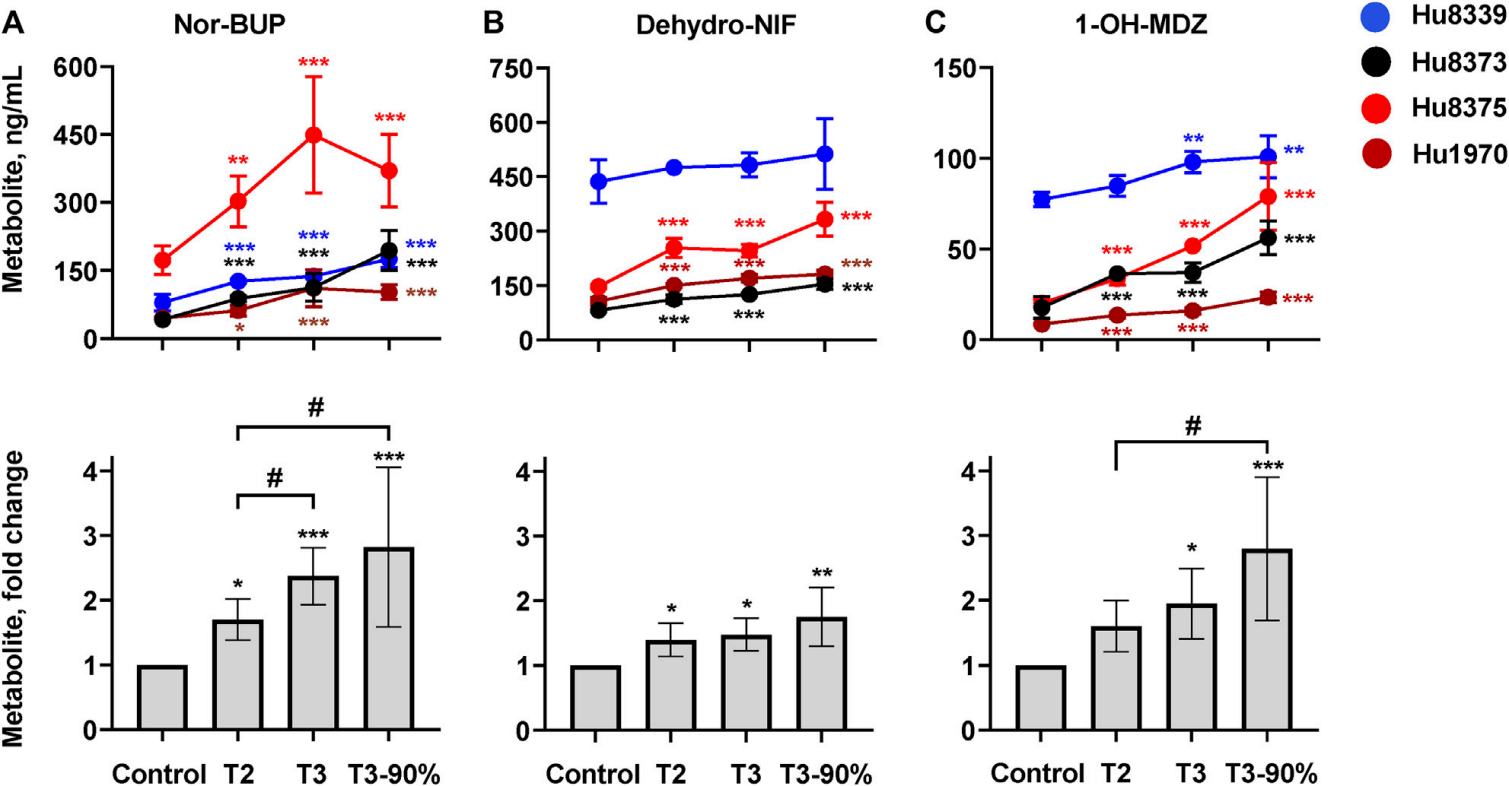
PRHs increased buprenorphine (BUP), nifedipine (NIF), and midazolam (MDZ) metabolism in sandwich-cultured human hepatocytes (SCHH). SCHH (*n* = 4 donors) were exposed to vehicle control or PRH cocktails targeting average trimester 2 (T2), average trimester 3 (T3), and upper range of T3 (T3-90%) for 72 h prior to incubation with CYP3A substrates BUP, NIF, or MDZ. The line graphs depict the mean ± SD formation of nor-BUP **(A)**, dehydro-NIF **(B)**, and 1-OH-MDZ **(C)** within each donor (*n* = 3–4 replicates per group; *n* = 2 replicates in the MDZ experiment T3 group in donor Hu8375 due to a concentration measurement analytical failure). The corresponding bar graphs exhibit the net mean ± SD fold-difference for each metabolite relative to vehicle control across all donors (*n* = 4 per group). *p < 0.05, **p < 0.01, ***p < 0.001 *versus* vehicle control. *#* Represents the concentration-dependent effect across PRH groups (#< 0.05).

We quantified and compared the impact of PRH exposure on the metabolism of the opioid BUP, antihypertensive NIF, and the probe substrate MDZ to their respective CYP3A-mediated metabolites, nor-BUP, dehydro-NIF, and 1-OH-MDZ in SCHH (Figure 3; Supplementary Table S5). PRHs significantly increased the formation of nor-BUP (Figure 3A), dehydro-NIF (Figure 3B), and 1-OH-MDZ (Figure 3C) relative to the vehicle control (ANOVA *p* < 0.05). The effects appeared to be PRH concentration-dependent, but the magnitude of the PRH-mediated induction in metabolism appeared to vary across substrate and hepatocyte donor.

#### Buprenorphine metabolism

PRH exposure increased nor-BUP formation relative to control in a concentration-dependent manner within each of the four hepatocyte donors and across all donors (ANOVA *p* < 0.001). Relative to control, the average net increase in nor-BUP formation across hepatocyte donors was 1.70 ± 0.32, 2.37 ± 0.44, and 2.82 ± 1.23-fold in the T2, T3, and T3-90% PRH groups, respectively (Figure 3A). The magnitude of the T3 PRH group effect on nor-BUP formation within each individual donor was 1.73 ± 0.06 (Hu8339), 2.70 ± 0.73 (Hu8373), 2.60 ± 0.74 (Hu8375) and 2.45 ± 0.91 (Hu1970) (Supplementary Table S5).

Under basal conditions, nor-BUP formation in CYP3A5 expresser donor Hu8339 appeared similar to the CYP3A5 non-expresser donors (Figure 3A). The PRH-evoked increase in nor-BUP formation ranged from 1.74 ± 0.38-fold (T2) to 3.02 ± 1.42-fold (T3-90%) in CYP3A5 non-expressers and from 1.58- to 2.20-fold, respectively, in the CYP3A5 expresser donor (Supplementary Table S5).

#### Nifedipine metabolism.

PRH exposure increased dehydro-NIF formation relative to control within three of the four hepatocyte donors and across all donors (ANOVA *p* = 0.013). Relative to control, the average net increase in dehydro-NIF formation across hepatocyte donors was 1.40 ± 0.26, 1.48 ± 0.26, and 1.76 ± 0.45-fold in the T2, T3, and T3-90% PRH groups, respectively (Figure 3B). The magnitude of the T3 PRH group effect on dehydro-NIF formation within each individual donor was 1.11 ± 0.08 (Hu8339), 1.54 ± 0.05 (Hu8373), 1.67 ± 0.12 (Hu8375) and 1.60 ± 0.09 (Hu1970) (Supplementary Table S5).

As depicted in Figure 3B, dehydro-NIF formation under basal conditions was 4.14 ± 1.20-fold higher in the CYP3A5 expresser donor Hu8339, on average, compared to the CYP3A5 non-expresser donors (Hu8373, Hu8375, and Hu1970). Similar to induction of total CYP3A protein, the impact of PRH exposure on dehydro-NIF formation was more pronounced in CYP3A5 non-expressers compared to CYP3A5 expresser donor Hu8339. The PRH increase in dehydro-NIF formation ranged from 1.50 ± 0.19 (T2) to 1.95 ± 0.28-fold (T3-90%) in the CYP3A5 nonexpressers and from 1.09- to 1.17-fold, respectively, in the CYP3A5 expresser donor (Supplementary Table S5).

#### Midazolam metabolism

PRH exposure increased 1-OH-MDZ formation relative to control in a concentration-dependent manner within each of the four hepatocyte donors and across all donors (ANOVA *p* = 0.007). Relative to control, the average net increase in 1-OH-MDZ formation across hepatocyte donors was 1.60 ± 0.40, 1.95 ± 0.54, and 2.80 ± 1.09-fold in the T2, T3, and T3-90% PRH groups, respectively (Figure 3C); however, the observed effect in the T2 PRH group was not statistically significant compared to control (*p* = 0.064). The magnitude of the T3 PRH group increase in 1-OH-MDZ formation within each individual donor was 1.27 ± 0.08 (Hu8339), 2.10 ± 0.30 (Hu8373), 2.57 ± 0.02 (Hu8375) and 1.87 ± 0.22 (Hu1970) (Supplementary Table S5).

As depicted in Figure 3C, basal formation of 1-OH-MDZ was 5.79 ± 2.93-fold higher in donor Hu8339 compared to the CYP3A5 non-expresser donors. Similar to induction of dehydro-NIF formation, the impact of PRH exposure on 1-OH-MDZ formation was more pronounced in CYP3A5 non-expressers compared to CYP3A5 expresser donor Hu8339. The PRH-evoked increase in 1-OH-MDZ formation ranged from 1.77 ± 0.25-fold (T2) to 3.29 ± 0.58-fold (T3-90%) in CYP3A5 non-expressers and from 1.10- to 1.31-fold, respectively, in the CYP3A5 expresser donor (Supplementary Table S5).

### PRH mediated increases in CYP3A protein concentrations correlate with changes in metabolism

**FIGURE 4:**
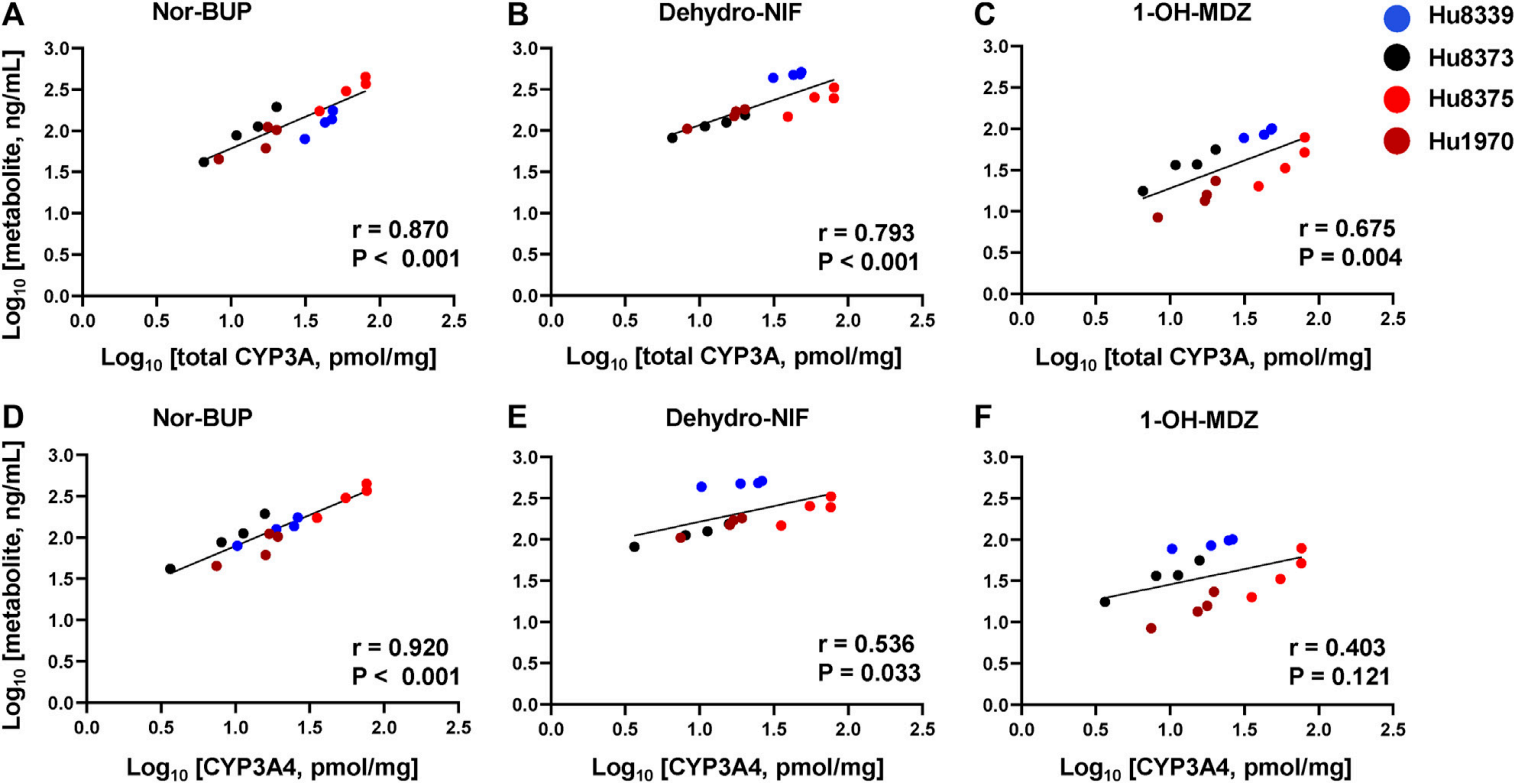
Correlation between total CYP3A or CYP3A4 protein concentration and metabolite formation in sandwich-cultured human hepatocytes (SCHH). Correlation between total CYP3A (CYP3A4 + 3A5 + 3A7) **(A–C)** and CYP3A4 **(D–F)** absolute protein concentrations with norbuprenorphine (nor-BUP) **(A,D)**, dehydro nifedipine (dehydro-NIF) **(B,E)**, and 1-OH-midazolam (1-OH-MDZ) concentration **(C,F)** in SCHH exposed to vehicle control or PRH cocktails (*n* = 4 donors). Each data point represents the mean concentration for each treatment group within each donor. The Pearson correlation coefficient (*r*) and *p*-values are provided.

In order to examine whether the observed PRH-evoked increase in nor-BUP, dehydro-NIF and 1-OH-MDZ formation was driven by the increase in CYP3A4 and total CYP3A protein concentrations, correlations between metabolite and protein concentrations across SCHH donors were evaluated (Figure 4). Formation of nor-BUP demonstrated a strong positive correlation with both total CYP3A absolute protein concentrations (*r* = 0.870, *p* < 0.001; Figure 4A) and CYP3A4 absolute protein concentrations (*r* = 0.920, *p* < 0.001; Figure 4D). Total CYP3A protein concentrations also positively and significantly correlated with the formation of dehydro-NIF (*r* = 0.793, *p* < 0.001; Figure 4B) and 1-OH-MDZ (*r* = 0.675, *p* = 0.004; Figure 4C). In contrast, the correlation strength between absolute CYP3A4 protein concentrations and the formation of dehydro-NIF (*r* = 0.536, *p* = 0.033; Figure 4E) and 1-OH-MDZ (*r* = 0.403, *p* = 0.121; Figure 4F) was lower compared to that observed with total CYP3A. Visual inspection of the correlation plots revealed that donor Hu8339 (a CYP3A5 expresser) appeared to contribute to the weakened correlations between CYP3A4 protein concentrations and dehydro-NIF and 1-OH-MDZ formation. Re-evaluation of correlations exclusively within the CYP3A5 non-expresser donors strengthened the association between CYP3A4 protein expression and formation of dehydro-NIF (*r* = 0.948, *p* < 0.001) and 1-OH-MDZ (*r* = 0.558, *p* = 0.059) (Supplementary Figure S2).

## Discussion

Accumulating evidence has shown that pregnancy increases clearance and reduces systemic exposure to CYP3A substrate drugs in humans (Pariente et al., 2016; Tasnif et al., 2016; Mulrenin et al., 2021). Moreover, *in vitro* studies have demonstrated that PRHs induce CYP3A4 expression and MDZ and NIF metabolism in human hepatocytes (Choi et al., 2013; Isoherranen and Thummel, 2013; Zhang et al., 2015; Khatri et al., 2021b). However, the impact of PRHs on CYP3A4-mediated metabolism of BUP has not been demonstrated. Additionally, it remains unknown whether the extent of pregnancy-associated changes in hepatic CYP3A4-mediated metabolism varies in a substrate-dependent manner. In this study, we quantified and compared the impact of PRHs on the absolute protein concentration of CYP3A isoforms (CYP3A4, 3A5, and 3A7) and the metabolism of CYP3A substrates BUP (opioid), NIF (antihypertensive), and MDZ (phenotyping probe) in SCHH derived from multiple donors (one of which was a CYP3A5 expresser). Our data illustrated that 1) PRHs increased BUP, NIF, and MDZ metabolism in a concentration-dependent manner by increasing hepatic CYP3A4 and total CYP3A protein concentrations, and 2) the magnitude of the PRH-evoked increase in nor-BUP, dehydro-NIF, and 1-OH-MDZ formation varied across hepatocyte donors in a substrate-dependent manner. Upon exploration of inter-donor differences, the magnitude of the PRH-mediated increase in NIF and MDZ metabolism, but not BUP metabolism, appeared to be mitigated in the hepatocyte donor with high basal CYP3A5 expression. These experimental data provide insight into the contribution of PRH-mediated induction of hepatic CYP3A4 expression and metabolism to the increases in BUP oral clearance observed during pregnancy in humans, and offer the potential to inform more precise dosing recommendations in pregnant patients treated with BUP.

Probe substrate studies in pregnant volunteers have shown that CYP3A metabolic activity, measured by 1-OH-MDZ formation clearance, urinary excretion of dextromethorphan/3-OH-dextromethorphan ratio, or plasma 4β-OH-cholesterol/cholesterol ratio, is increased by approximately 1.5–2.0-fold during pregnancy (Tracy et al., 2005; Hebert et al., 2008; Kim et al., 2018; Mlugu et al., 2022). Because it is well-established that hepatic CYP3A protein concentration and metabolite formation are directly related (Kronbach et al., 1989; Wolbold et al., 2003), the central hypothesis has been that the gestational surge in PRHs induces *CYP3A4* transcriptional regulation via nuclear receptor activation, and subsequent increases in CYP3A4 protein expression in hepatocytes drive increases in CYP3A4 metabolism (Jeong, 2010). Consistent with this hypothesis, previous studies have reported that PRHs such as E2, P4, and CRT administered individually or in combination induce *CYP3A4* mRNA levels, CYP3A4 protein expression, and dehydro-NIF and 1-OH-MDZ formation in human hepatocytes (Choi et al., 2013; Papageorgiou et al., 2013; Zhang et al., 2015; Khatri et al., 2021b). Our results extend these prior observations with QTAP based quantification of absolute CYP3A isoform concentrations in a larger number of hepatocyte donors, and demonstrate that PRH exposure does not significantly induce CYP3A5 protein, modestly induces CYP3A7, and yields a concentration-dependent increase in total CYP3A protein concentrations within and across donors via induction of CYP3A4. The observed isoform-specific induction by PRHs was similar to the prototypical CYP3A inducer RIF, which yielded negligible induction of CYP3A5 protein compared to CYP3A4. Although increased *CYP3A4* transcriptional activation has been established as a mechanism underlying these PRH effects (Sachar et al., 2019), future studies remain necessary to discern whether altered posttranslational modification also is a mechanism that contributes to PRH-mediated increases in hepatic CYP3A4 protein concentrations.

Clinical pharmacokinetic and PBPK modeling studies have demonstrated that BUP, NIF, and MDZ oral clearance are increased by approximately 1.5–2.0-fold during T3, on average, and suggest that these effects are mediated in large part via a pregnancy-associated increase in hepatic CYP3A-mediated metabolism; however, substantial inter-patient variability exists (Hebert et al., 2008; Ke et al., 2012; Dallmann et al., 2018a; Zhang et al., 2018; Zhang et al., 2020; Mulrenin et al., 2021). In our experiments, exposure to PRHs targeting T3 concentrations increased total CYP3A protein concentration (1.94 ± 0.32) and nor-BUP (2.37 ± 0.44), dehydro-NIF (1.48 ± 0.26), and 1-OH-MDZ (1.95 ± 0.54) formation, relative to control, across hepatocyte donors. Moreover, total CYP3A protein concentrations positively correlated with nor-BUP, dehydro-NIF, and 1-OH-MDZ formation. These results provide experimental evidence in support of PBPK model predicted increases in hepatic CYP3A-mediated BUP, NIF, and MDZ metabolism and clearance during human pregnancy, and offer new insight into the contribution of PRH-mediated induction of hepatic CYP3A4 to the pregnancy-associated increases in BUP clearance.

Our experiments also illustrated that the magnitude of PRH-evoked increases in CYP3A-mediated metabolism varied across hepatocyte donors and substrates, and suggested that basal CYP3A5 expression and substrate-specific differences in the relative contributions of CYP3A4 and CYP3A5 may influence the presence and magnitude of PRH induction of hepatic CYP3A metabolism. In adults, hepatic CYP3A activity collectively reflects the net metabolism that occurs by CYP3A4 and CYP3A5, which share 84% amino acid sequence homology and exhibit overlap in substrate metabolism (Wilkinson, 2005; Tseng et al., 2014; Lolodi et al., 2017). In contrast to CYP3A4, CYP3A5 is highly polymorphic and exhibits complete absence of expressed functional enzyme due to the CYP3A5*3, *6 and *7 no function alleles (Kuehl et al., 2001; Birdwell et al., 2015). The minor allele frequencies of CYP3A5*3, *6 and *7 vary considerably across populations, and are approximately 25%–30%, 10%–20% and 8%–12% in populations of African ancestry and 90%, 0.2% and 0% in populations of European ancestry; together, these alleles yield absence of CYP3A5 expression and activity in approximately 30% of African and 85% of European populations (Roy et al., 2005; Birdwell et al., 2015). Probe substrate studies in African-American volunteers have reported higher CYP3A metabolic activity in CYP3A5 expressers compared in non-expressers (Roberts et al., 2008), and a pharmacokinetic study in 14 preterm labor patients reported approximately 2.5-fold higher NIF oral clearance and lower NIF systemic exposure in CYP3A5 expressers compared to non-expressers (Haas et al., 2013). Consistent with ancestral differences in allele frequency, our QTAP analysis confirmed that African-American hepatocyte donor Hu8339 was a CYP3A5 expresser and the remaining White donors were CYP3A5 non-expressers. The PRH induction of CYP3A4 protein expression and nor-BUP formation appeared to be comparable in the CYP3A5 expresser and non-expressers; in contrast, the magnitude of the PRH-mediated increase in total CYP3A protein concentrations and formation of dehydro-NIF and 1-OH-MDZ appeared to be diminished in the CYP3A5 expresser donor compared to the non-expressers.

Differences in the relative contribution of CYP3A4 and CYP3A5 mediated intrinsic clearance may help explain the observed influence of basal CYP3A5 expression on substrate differences in basal and PRH-induced changes in MDZ and NIF metabolism, but not BUP metabolism. An *in vitro* study in liver microsomes from CYP3A5*1/*1 expressers in the presence of ketoconazole (CYP3A inhibitor) confirmed that CYP3A4/5 enzymes mediate over 90% and 95% of 1-OH-MDZ and dehydro-NIF formation (Tseng et al., 2014). Experiments from the same study using CYP3cide (CYP3A4 inhibitor) in CYP3A5 expressers showed that approximately 50:50% of 1-OH-MDZ and 81:14% of dehydro-NIF formation was attributed specifically to CYP3A4:3A5 activity, respectively; whereas, >95% of 1-OH-MDZ and dehydro-NIF formation was mediated by CYP3A4 in microsomes from CYP3A5*3/*3 non-expressers (Tseng et al., 2014). Enzyme kinetics experiments have similarly demonstrated that 1-OH-MDZ intrinsic clearance is approximately equivalent in recombinant CYP3A4 *versus* CYP3A5 (0.5–2.0 fold relative difference), whereas dehydro-NIF intrinsic clearance is approximately 10–20-fold higher in recombinant CYP3A4 (Williams et al., 2002; Patki et al., 2003; Tseng et al., 2014). In contrast, CYP3A5 is a minor contributor (<5%) to nor-BUP formation. A study in liver microsomes in the presence of ketoconazole (CYP3A inhibitor) and trimethoprim (CYP2C8 inhibitor) demonstrated that nor-BUP formation is predominantly driven by CYP3A4 (65%) and to a lesser extent CYP2C8 (30%) (Picard et al., 2005). Enzyme kinetics experiments have similarly demonstrated that nor-BUP intrinsic clearance is approximately 3-fold higher in recombinant CYP3A4 *versus* CYP2C8 protein (Picard et al., 2005). The significant contributions of CYP3A5 activity to MDZ and NIF, but not BUP, metabolism in these prior *in vitro* studies are consistent with our observation that basal 1-OH-MDZ and dehydro-NIF formation, but not nor-BUP formation, was several-fold higher in donor Hu8339 (CYP3A5*1/*1) relative to the CYP3A5 non-expresser donors. In addition, given the absence of CYP3A5 induction by PRHs, the PRH-mediated increase in 1-OH-MDZ and dehydro-NIF formation more strongly correlated with total CYP3A than CYP3A4 protein concentrations in our experiments; in contrast, nor-BUP formation correlations with total CYP3A and CYP3A4 concentrations were similarly very strong. Together, these data confirm that CYP3A-mediated BUP metabolism is predominantly driven by CYP3A4 and is less sensitive to inter-individual differences in basal CYP3A5 expression than MDZ and NIF metabolism.

In addition to CYP3A4, CYP2C8 may also contribute to the observed PRH-evoked increase in nor-BUP formation. Induction of CYP2C8 by PRHs in human hepatocytes has been previously reported; however, the magnitude of induction was less than CYP3A4 (Choi et al., 2013; Khatri et al., 2021b). In addition, functional studies with a CYP2C8 probe substrate have not been completed to confirm that PRHs induce CYP2C8-mediated metabolism in human hepatocytes. Given the modest induction of CYP2C8 protein concentrations in our experiments (an effect was negligible in multiple hepatocyte donors), and the relatively minor contribution of CYP2C8 to nor-BUP formation (Picard et al., 2005), the contribution of CYP2C8 induction to the observed PRH-mediated increases in nor-BUP formation in our experiments was likely negligible. In addition to nor-BUP formation, both BUP and nor-BUP undergo glucuronidation by UGT1A1, UGT1A3, and UGT2B7 (Rouguieg et al., 2010). In human hepatocytes, PRHs increase UGT1A1 protein concentrations in a donor-specific manner, but do not alter UGT1A3 and UGT2B7 protein concentration (Khatri et al., 2021a; Fashe et al., 2022). Future studies are warranted to elucidate the impact of PRHs on UGT1A1-mediated glucuronidation of BUP and nor-BUP.

The impact of *CYP3A5* genotype and expresser status on pregnancy-associated changes in CYP3A metabolism requires further study. A recent study in Tanzanian pregnant women showed that the plasma 4β-OH-cholesterol/cholesterol ratio did not significantly differ in CYP3A5 expressers and non-expressers (Mlugu et al., 2022). Similar to nor-BUP, this may be explained by a minor contribution of CYP3A5 to 4β-OH-cholesterol formation *in vitro* (Bodin et al., 2002). Although it is important to note that our *in vitro* data were exploratory, derived from a single CYP3A5 expresser donor, and thus should be interpreted with caution, our data suggest that basal CYP3A5 expression status may modify the magnitude of PRH effects on the induction of total CYP3A protein concentrations and hepatic CYP3A metabolism in a substrate-dependent manner. This observation is consistent with a study in African-American volunteers demonstrating that dexamethasone treatment significantly induced CYP3A activity, as measured by the erythromycin breath test, in CYP3A5 non-expressers but did not increase activity in CYP3A5 expressers (Roberts et al., 2008). Taken together, these results illustrate the need for future PRH *in vitro* metabolism experiments in hepatocytes derived from multiple racially diverse donors, and *in vivo* pregnancy pharmacokinetic studies in more racially diverse populations, in order to elucidate the impact of *CYP3A5* genotype on pregnancy-related increases in hepatic CYP3A metabolism and clearance across multiple CYP3A substrates.

## Conclusion

To our knowledge, this is the first study to quantify and compare the impact of PRHs on CYP3A absolute protein concentrations and the metabolism of BUP in human primary hepatocytes, and to directly compare the magnitude of these effects to the CYP3A substrates NIF and MDZ. PRHs induced a significant net increase in nor-BUP, dehydro-NIF and 1-OH-MDZ formation across hepatocyte donors by inducing absolute CYP3A4 and total CYP3A protein concentrations. However, differences in the magnitude of these effects were observed across substrates. While the increase in CYP3A4 expression and nor-BUP formation was evident in all donors, increases in dehydro-NIF and 1-OH-MDZ formation were most prominently observed in CYP3A5 non-expressers and appeared to be lessened in the CYP3A5 expresser donor. Collectively, these data provide experimental validation of previous clinical observations predicted by PBPK models that BUP, NIF, and MDZ clearance is increased by 1.5–2.0-fold during pregnancy due to increased hepatic CYP3A4-mediated metabolism (Ke et al., 2012; Zhang et al., 2018), and suggest that basal CYP3A5 expression may modify pregnancy-associated increases in hepatic CYP3A metabolism in a substrate-specific manner.

## Figures and Tables

**Table:** 

**1-OH-MDZ**	1-hydroxymidazolam
**ACN**	Acetonitrile
**BUP**	Buprenorphine
**CRT**	Cortisol
**dehydro-NIF**	Dehydronifedipine
**DME**	Drug metabolizing enzyme
**DMSO**	Dimethyl sulfoxide
**E1**	Estrone
**E2**	Estradiol
**E3**	Estriol
**EDTA**	Ethylenediaminetetraacetic acid
**FA**	Formic acid
**HDP**	Hypertensive disorders of pregnancy
**LC-MS**	Liquid chromatography—mass spectrometry
**LLOQ**	Lower limit of quantitation
**MDZ**	Midazolam
**NIF**	Nifedipine
**nor-BUP**	Norbuprenorphine
**OUD**	Opioid use disorder
**P4**	Progesterone
**PBPK**	Physiologically-based pharmacokinetic
**pGH**	Placental growth hormone
**PIPES**	Piperazine-N,N′-bis(2-ethanesulfonic acid)
**PMSF**	Phenylmethylsulfonyl fluoride
**PRHs**	Pregnancy related hormones
**QTAP**	Quantitative targeted absolute proteomics
**RIF**	Rifampicin
**SCHH**	Sandwich-cultured human hepatocytes
**SIL**	Stable isotope labeled
**T2**	Trimester 2 average
**T3**	Trimester 3 average
**T3-90%**	Trimester 3 90th percentile

## Data Availability

The raw data supporting the conclusion of this article will be made available by the authors, without undue reservation.
