# Supplementary material for: Pregnancy related hormones increase CYP3A mediated buprenorphine metabolism in human hepatocytes: a comparison to CYP3A substrates nifedipine and midazolam
Source: Front Pharmacol. 2023 Jul 5;14:1218703. doi: 10.3389/fphar.2023.1218703 (PMC10354249; doi:10.3389/fphar.2023.1218703)
Supplement: Supplementary file 1 [file DataSheet1.PDF]

Suppl. Table S1. Primary hepatocyte female donor characteristics.

Donors	Age (yr.)	Race	Vendor	Transporter qualified	Induction qualified	Drug history	Cause of Death	CYP3A5 (*3)
**Hu8339**	31	African American	Life Technologies	Yes	Yes	Marijuana	Asphyxiation	WT/WT
**Hu8373**	26	Caucasian	Life Technologies	No	Yes	Cocaine, cannabis	Asphyxiation	**3/*3*
**Hu8375**	19	Caucasian	Life Technologies	Yes	Yes	Cannabis	Asphyxiation	**3/*3*
**YNM**	48	Caucasian	BioIVT	Yes	Yes	Not reported	Anoxia	Not reported [a]
**Hu1970**	34	Caucasian	Life Technologies	Yes	Yes	Not reported	Not reported	**3/*3*

[a] *Although CYP3A5 genotype was not reported, basal CYP3A5 protein concentrations were <1 pmol/mg protein in this donor and the donor is considered a CYP3A5 non-expresser*

Suppl. Table S2. Pregnancy related hormone (PRH) concentrations exogenously administered to hepatocytes.

**Treatment Group**	**E1 (nM)**	**E2 (nM)**	**E3 (nM)**	**P4 (nM)**	**CRT (nM)**	**pGH (nM)**
**T2**	125	225	125	1000	800	0.35
**T3**	250	500	250	2500	800	1.34
**T3-90%**	450	750	450	3750	1300	3.13

As previously reported in detail (Fashe et al., 2022), the listed concentrations (nM) are the concentrations of each PRH exogenously administered in combination as a cocktail to the sandwich cultured human hepatocyte medium in the experimental model. Based on previously estimated elimination half-life estimates of each PRH in cultured human hepatocytes, these are the PRH treatment concentrations needed to achieve the desired concentration of each PRH in our hepatocyte model system that target the average trimester 2 (T2), average trimester 3 (T3), and upper range of T3 (T3-90%) circulating concentrations in human pregnancy.

Reference: Fashe, M.M., Fallon, J.K., Miner, T.A., Tiley, J.B., Smith, P.C., and Lee, C.R. (2022). Impact of pregnancy related hormones on drug metabolizing enzyme and transport protein concentrations in human hepatocytes. *Front Pharmacol* 13**,** 1004010. doi: 10.3389/fphar.2022.1004010.

Suppl. Table S3. Impact of pregnancy related hormones on CYP3A protein concentrations in SCHH

	**Hu8339**	**Hu8373**	**Hu8375**	**YNM**	**Hu1970**	**Combined [a] (All Donors)**	**Combined [a] (without Hu8339)**
**CYP3A4**							
**Control**	1.00 ± 0.11	1.00 ± 0.38	1.00 ± 0.16	1.00 ± 0.02	1.00 ± 0.14	1.00	1.00
**T2**	1.83 ± 0.27*	2.21 ± 0.64*	1.56 ± 0.25*	1.85 ± 0.11*	2.14 ± 0.18*	1.92 ± 0.26*	1.94 ± 0.30*
**T3**	2.41 ± 0.45*	3.11 ± 0.98*	2.15 ± 0.34*	1.79 ± 0.18*	2.27 ± 0.23*	2.34 ± 0.48*	2.33 ± 0.56*
**T3-90**	2.55 ± 0.33*	4.34 ± 1.06*	2.16 ± 0.08*	2.60 ± 0.44*	2.58 ± 0.25*	2.84 ± 0.85*	2.92 ± 0.97*
ANOVA P	*< 0.001*	*< 0.001*	*< 0.001*	*< 0.001*	*< 0.001*	*< 0.001*	*< 0.001*
**CYP3A5**							
**Control**	1.00 ± 0.04	1.00 ± 0.23	1.00 ± 0.16	1.00 ± 0.19	1.00 ± 0.29	1.00	1.00
**T2**	1.15 ± 0.12	0.75 ± 0.29	0.77 ± 0.14	1.29 ± 0.39	0.89 ± 0.38	0.97 ± 0.24	0.92 ± 0.25
**T3**	1.03 ± 0.10	0.94 ± 0.63	0.78 ± 0.10	0.78 ± 0.39	0.68 ± 0.17	0.84 ± 0.14	0.80 ± 0.11
**T3-90**	1.01 ± 0.07	0.76 ± 0.27	0.71 ± 0.17	0.37 ± 0.34	0.89 ± 0.34	0.74 ± 0.24	0.68 ± 0.22
ANOVA P	*0.113*	*0.704*	*0.123*	*0.196*	*0.570*	*0.159*	*0.118*
**CYP3A7**							
**Control**	1.00 ± 0.16	1.00 ± 0.21	1.00 ± 0.09	1.00 ± 0.98	1.00 ± 0.12	1.00	1.00
**T2**	0.99 ± 0.19	1.01 ± 0.18	1.46 ± 0.34	1.26 ± 0.37	2.34 ± 0.72*	1.41 ± 0.55*	1.52 ± 0.58
**T3**	1.66 ± 0.25*	1.40 ± 0.14*	1.36 ± 0.35	1.43 ± 0.49	1.47 ± 0.38*	1.46 ± 0.12*	1.42 ± 0.05
**T3-90**	1.47 ± 0.27*	1.68 ± 0.33*	1.44 ± 0.20	1.50 ± 1.36	2.07 ± 0.87*	1.63 ± 0.26*	1.67 ± 0.29
ANOVA P	*0.002*	*0.004*	*0.081*	*0.791*	*0.029*	*0.008*	*0.020*
**Total CYP3A (3A4+3A5+3A7)**							
**Control**	1.00 ± 0.07	1.00 ± 0.28	1.00 ± 0.15	1.00 ± 0.06	1.00 ± 0.12	1.00	1.00
**T2**	1.37 ± 0.11*	1.66 ± 0.35*	1.51 ± 0.24*	1.79 ± 0.13*	2.05 ± 0.15*	1.68 ± 0.27*	1.76 ± 0.24*
**T3**	1.52 ± 0.21*	2.31 ± 0.60*	2.04 ± 0.32*	1.71 ± 0.15*	2.11 ± 0.19*	1.94 ± 0.32*	2.05 ± 0.25*
**T3-90**	1.54 ± 0.15*	3.08 ± 0.67*	2.04 ± 0.07*	2.42 ± 0.45*	2.42 ± 0.21*	2.31 ± 0.57*	2.50 + 0.43*
ANOVA P	*< 0.001*	*< 0.001*	*< 0.001*	*< 0.001*	*< 0.001*	*< 0.001*	*< 0.001*

*Data presented as mean ± SD. * p < 0.05 versus control group*

[a] *Combined was calculated with and without fold change data from hepatocyte donor Hu8339 (CYP3A5 expresser).*

Suppl. Table S4. Impact of pregnancy related hormones on CYP2C8 protein concentrations in SCHH

	**Hu8339**	**Hu8373**	**Hu8375**	**YNM**	**Hu1970**	**Combined (All Donors)**
**CYP2C8**						
**Control**	1.00 ± 0.46	1.00 ± 0.11	1.00 ± 0.21	1.00 ± 0.37	1.00 ± 0.17	1.00
**T2**	1.23 ± 0.10	1.49 ± 0.23	1.13 ± 0.19	1.23 ± 0.57	1.43 ± 1.62	1.30 ± 0.15
**T3**	1.15 ± 0.18	1.32 ± 0.19	1.05 ± 0.03	0.81 ± 0.33	0.97 ± 0.7	1.06 ± 0.19
**T3-90**	1.23 ± 0.20	2.18 ± 0.49	1.20 ± 0.12	1.95 ± 0.69	1.15 ± 1.09	1.54 ± 0.49
ANOVA P	*0.368*	*< 0.001*	*0.343*	*0.132*	*0.920*	*0.014*

*Data presented as mean ± SD. * p < 0.05 versus control group*

Suppl. Table S5. Impact of pregnancy related hormones on midazolam, nifedipine, and buprenorphine metabolism in SCHH

	Hu8339	Hu8373	Hu8375	Hu1970	Combined [a] (All Donors)	Combined [a] (without Hu8339)
**Norbuprenorphine**						
**Control**	1.00 ± 0.05	1.00 ± 0.11	1.00 ± 0.01	1.00 ± 0.05	1.00	1.00
**T2**	1.58 ± 0.06*	2.11 ± 0.16*	1.75 ± 0.32*	1.36 ± 0.25*	1.70 ± 0.32*	1.74 ± 0.38*
**T3**	1.73 ± 0.06*	2.70 ± 0.73*	2.60 ± 0.74*	2.45 ± 0.91*	2.37 ± 0.44*	2.59 ± 0.13*
**T3-90**	2.20 ± 0.24*	4.66 ± 1.06*	2.14 ± 0.46*	2.26 ± 0.37*	2.82 ± 1.23*	3.02 ± 1.42*
ANOVA P	*< 0.001*	*< 0.001*	*0.002*	*< 0.001*	*< 0.001*	*0.003*
**Dehydro nifedipine**						
**Control**	1.00 ± 0.14	1.00 ± 0.23	1.00 ± 0.03	1.00 ± 0.09	1.00	1.00
**T2**	1.09 ± 0.03	1.38 ± 0.15*	1.72 ± 0.18*	1.41 ± 0.05*	1.40 ± 0.26*	1.50 ± 0.19*
**T3**	1.11 ± 0.08	1.54 ± 0.05*	1.67 ± 0.12*	1.60 ± 0.09*	1.48 ± 0.26*	1.60 ± 0.06*
**T3-90**	1.17 ± 0.22	1.89 ± 0.18*	2.26 ± 0.32*	1.70 ± 0.10*	1.76 ± 0.45*	1.95 ± 0.28*
ANOVA P	*0.413*	*< 0.001*	*< 0.001*	*< 0.001*	*0.013*	*< 0.001*
**1-OH-midazolam**						
**Control**	1.00 ± 0.05	1.00 ± 0.34	1.00 ± 0.09	1.00 ± 0.05	1.00	1.00
**T2**	1.10 ± 0.08	2.07 ± 0.12*	1.66 ± 0.17*	1.59 ± 0.17*	1.60 ± 0.40	1.77 ± 0.25*
**T3**	1.27 ± 0.08*	2.10 ± 0.30*	2.57 ± 0.02*	1.87 ± 0.22*	1.95 ± 0.54*	2.18 ± 0.35*
**T3-90**	1.31 ± 0.15*	3.19 ± 0.53*	3.92 ± 0.93*	2.77 ± 0.35*	2.80 ± 1.09*	3.29 ± 0.58*
ANOVA P	*0.001*	*< 0.001*	*< 0.001*	*< 0.001*	*0.007*	*< 0.001*

*Data presented as mean ± SD. * p < 0.05 versus control group*

[a] *Combined was calculated with and without fold change data from hepatocyte donor Hu8339 (CYP3A5 expresser).*

Suppl. Figure S1

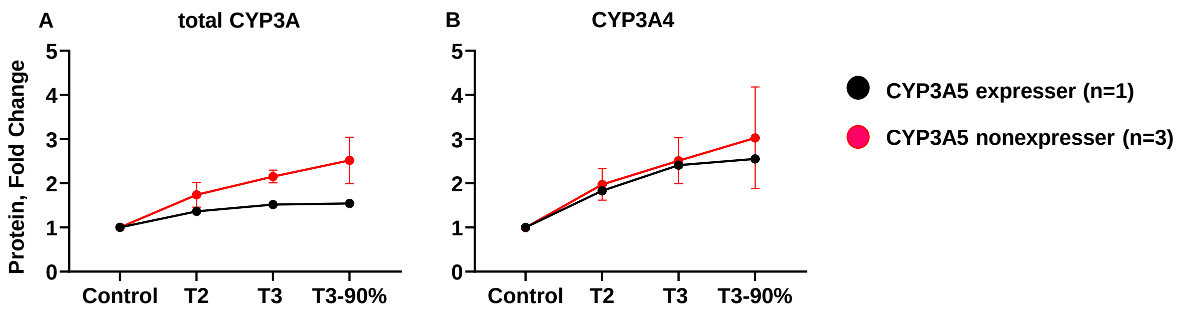

Supplemental Figure S1. *Impact of PRHs on CYP3A absolute protein concentration and substrate metabolism in CYP3A5 expressers and non-expressers.* The line graphs depict mean ± SD fold-difference in total CYP3A protein concentration (**A**) and CYP3A4 protein concentration (**B**), relative to vehicle control, in CYP3A5 expressers (n = 1 donor; Hu8339) and non-expressers (n = 3 donors; Hu8373, Hu8375, Hu1970) exposed to vehicle control or PRH cocktails targeting average trimester 2 (T2), average trimester 3 (T3), or upper range of T3 (T3-90%).

Suppl. Figure S2.

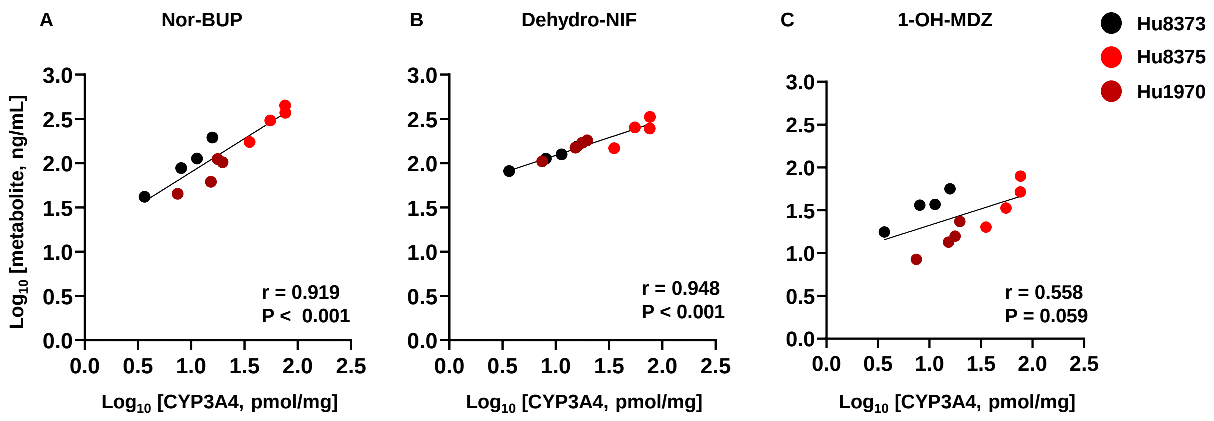

Supplemental Figure S2. *Correlation between CYP3A4 protein concentration and induction of metabolite formation in CYP3A5 non-expressers.* Correlation between CYP3A4 absolute protein concentration and norbuprenorphine (nor-BUP) (**A**), dehydro nifedipine (dehydro-NIF) (**B**), or 1-OH-midazolam (1-OH-MDZ) concentration (**C**) in SCHH (n = 3 CYP3A5 non-expresser donors) exposed to vehicle control or PRH cocktails. Each data point on the y-axis represents the mean concentration for each treatment group within each donor. The Pearson correlation coefficient (r) and p-values are provided.
